# Histopathology-based prognostic score is independent prognostic factor of gastric carcinoma

**DOI:** 10.1186/1471-2407-14-663

**Published:** 2014-09-11

**Authors:** Zhi Zhu, Xuren Sun, Jinou Wang, Zhe Sun, Zhenning Wang, Xinyu Zheng, Huimian Xu

**Affiliations:** Department of Surgical Oncology, First Affilated Hospital, China Medical University, North Nanjing Street 155, Shenyang, 110001 PR, China; Department of General Surgery, First Affiliated Hospital, China Medical University, Shenyang, China; Department of Gastroenterology, First Affiliated Hospital, China Medical University, Shenyang, China; Department of Pathology, Shengjing Hospital of China Medical University, Shenyang, China

**Keywords:** Gastric cancer, Histological type, Tumor differentiation, Prognosis

## Abstract

**Background:**

The aim of our study was to evaluate the histological characteristics and prognosis of gastric cancer.

**Methods:**

Clinicopathlogical variables of 932 patients with gastric carcinoma admitted to the Department of Surgical Oncology at the First Hospital of China Medical University were analyzed retrospectively. Different histological characteristics of gastric cancer were summarized and assigned score according to the malignancy defined by WHO classification, the scores were stratified into 4 stage, the prognosis of different stages were analyzed by Kaplan-Meier analysis and cox regression.

**Results:**

Among the 932 patients, 246 (26.39%) had mixed histology type of gastric cancer. Compared to the pure histological type, mixed histological type of gastric cancer was significant associated with tumor size, lymph node metastasis and depth of invasion (all P < 0.05). The 5-year survival rates of advanced and early gastric cancer patients with mixed type were 40.8% and 83.5% respectively, which were lower than those with pure type (50.0% and 95.8%, P < 0.01). Statistically significant difference with stratification of early and advanced stage could be observed between patients with the histological grading score. The data showed that the histological score could be the independent factor of prognosis.

**Conclusions:**

The histological score is an independent factor of gastric cancer, it exerts an excellent ability to classify survival of patients with gastric carcinoma. It also provides a new strategy and parameter for evaluating the biological behavior and prognosis of gastric cancer.

## Background

It might be easily thought out that tumor-related factors such as tumor size, lymphatic invasion, and venous invasion could be an indicator for progressive potential of malignant tumors [1–3]. However, except for stage of the tumor, there have not been any criteria using histopathological tumor-related factors to determine the outcome of the patients with gastric carcinoma.

Histopathological type is an important prognosis factor, it is also to determine the extent of surgical resection and formulated the basis for reasonable surgical plan [4, 5]. It plays an important role in prognostic score for a variety of tumors, such as Gleason score for prostate cancer [6], Child-pugh classification for hepatocellular carcinoma [7], SBR, WHO score for breast cancer [8], but the prognostic effect of histopathology has never been reported in gastric cancer. Moreover, there is not any effective way to identify the prognosis of early gastric cancer.

The coexistence of different histological types of gastric cancer determines the the complex characteristics of clinical behavior and prognosis, the mixed histological type accounted for over 25% of all gastric cancer [9, 10]. Since major diagnostic principles are used, the highly heterogeneous histological features of gastric cancer were ignored, tumor biological behavior and prognostic value of minor histological type were coved up [11]. We currently evaluated the prognostic significance of WHO histological classification of gastric cancer with a new histological scoring method, which makes the histopathological variable to be an independent prognostic factor.

In this study, we attempted to evaluate the histological characteristics and establish simple criteria using the results of histopathological tumor-related factors to predict prognosis of patients with gastric carcinoma.

### Methods

All patients with gastric cancer who underwent surgery at the Department of Surgical Oncology, First Hospital of China Medical University, during January 1980 to December 2006 were entered into a prospectively maintained database. Ethical approval for this research was obtained from the Research Ethics Committee of China Medical University, China. In total, 1077 patients underwent D2 lymphadenectomy and achieved radical (R0) resection for histologically proven gastric carcinoma. Follow-up was complete for the entire study population to June 2005. Among them, 24 died in the postoperative period and 43 were lost to follow-up. Thus, 145 patients were excluded. Of the remaining 932 patients, Median and mean follow-up period were 31 and 54 months (range: 3–313 months), respectively. Patients were treated exclusively by total or subtotal gastrectomy with lymphadenectomy, according to tumor location, adjuvant therapy or postoperative chemotherapy was not administered to any patient. Cancer-specific survival was calculated from the date of primary surgical resection to the date of gastric cancer associated death or to the date of recorded cancer progression. Tumor invasion (T), lymph node involvement (N) and TNM stage were classified according to the 7^th^ UICC/AJCC (2012) staging systems.

### Histological grading

**Figure 1:**
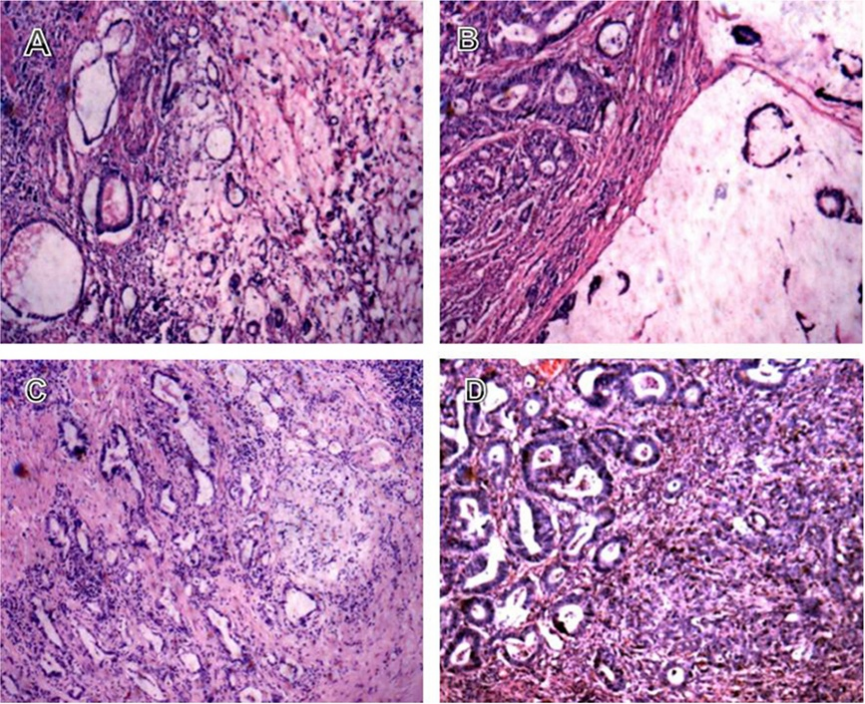
**Mixed histological type of gastric cancer in WHO classification. (A)** high differentiated tubular adenocarcinoma mixed with mucinous adenocarcinoma, final score: (1 + 3)/2 = 2, **(B)** medium differentiated tubular adenocarcinoma mixed with mucinous adenocarcinoma, final score: (2 + 3)/2 = 2.5 **(C)** high differentiated tubular adenocarcinoma mixed with signet ring cell carcinoma and mucinous adenocarcinoma, final score: (1 + 3 + 4)/3 = 2.7, **(D)** high differentiated mixed with undifferentiated gastric cancer, final score: (1 + 5)/2 = 3.

According to highly heterogeneous histological features and WHO classification of gastric cancer, we propose the novel histological grading system. Aiming to develop and validate the scoring system, the following statistical computing methodologies were applied in the order indicated:

1. According to the previous studies of histological differentiation and malignant degree of gastric carcinoma, non-mucinous adenocarcinoma (papillary adenocarcinoma and high, medium and low differentiated tubular adenocarcinoma) were assigned as 1–3 points, respectively. Mucinous adenocarcinoma was assigned as 3 points, signet ring cell carcinoma was assigned as 4 points, undifferentiated carcinoma was assigned as 4 points, a special type of stomach cancer as 4 points (these score still needs further refinement) (Table  1).(2) The total score of gastric carcinoma was summed by the original score of the primary histological type and secondary type of gastric carcinoma. Then the total score was divided by the number of histological types to calculate the final score (The mean of the total score). If it was composed of three or more histological type of gastric tissues, The scores will aggregate all score of histological type, then divideded by the number of histological types to get the final score, as presented in Figure  1. If the carcinoma was composed of a pure histological type (All tumors were constituted by the primary type), the score remains the final score.

**Table 1:** **Histological grading points of different subtypes of gastric carcinoma**

Gastric carcinoma	Score
Papillary carcinoma	1
Tubular carcinoma	
Well-differentiated	1
Medium-differentiated	2
Poorly-differentiated	3
Mucinous gastric carcinoma	3
signet-ring cell	4
Undifferentiated gastric carcinoma	5
Special type gastric carcinoma	4
Adeno-squamous carcinoma	
Squamous cell carcinoma	
Sarcomatoid type gastric cancer	
Liver adenocarcinoma	
Micro papillary carcinoma	
Neuroendocrine carcinoma	
Eosinophilic cell carcinoma

(3) Histological grading stage (H Stage) was divided into four groups according to their scores, as follows: H1 (≤2 points), H2 (2–3 points), H3 (3–4 points) and H4 (4–5 points).

### Statistical analysis

Each variable was used as an independent variable in a Cox proportional hazards analysis run using the Kaplan-Meier method. This was done to screen the variables for later inclusion in a multivariable model. To be included in the multivariable analysis, a variable had to be significant at α of 0.1 for the Wald test. This was a test of the null hypothesis that the coefficient for all levels of the variable of interest was equal to zero.

All significant variables of the univariate analysis were included in the first run of a multivariable Cox proportionate hazard regression. In subsequent Cox regressions, significantly different variables in their hazard ratios were remained sequentially based on the z-statistic for the individual levels of each categorical variable. To remain in the final model, a variable had to be significant at α of 0.05 at all levels.

## Results

As stated above, the study included 932 patients undergoing gastrectomy for gastric carcinoma from 1980 to 2005. All patients followed until death or for a maximum 313 months. The overall 5-year survival rate was 41.1%. Adjuvant and chemotherapy treatment was rarely applied in this series. The frequencies of patient characteristics and epidemiological results are summarized in Table  2.

**Table 2:** **Univariate analysis of the prognostic factors for patients with gastric cancer in overall and early stage**

Variables	Overall				Early stage			
	Cases	n(%)	5-year OS(%)	P value	Cases	n(%)	5-year OS(%)	P value
Gender	932			0.311	90		82.2	0.07
Male	669	71.8	37.8		65	72.2	86.2	
Female	263	28.2	43.1		25	27.8	72.0	
Age(years)				0.062				0.32
≤65	533	57.2	34.3		66	73.3	83.3	
>65	399	42.8	43.0		24	26.7	79.2	
Size				<0.001				0.714
≤4 cm	224	24.0	59.2		57	63.3	82.5	
>4 cm	708	76.0	33.0		33	36.7	81.8	
Location				<0.001				0.149
Lower	672	72.1	42.1		75	83.3	85.3	
Middle	99	10.6	37.6		9	10.0	66.7	
Upper	115	12.3	36.5		6	6.7	66.7	
Entire	46	4.9	25.4		0	0.0	0.0	
Macroscopic Type				<0.001				
Early stage	90	10.7	81.0					
Borrmann I	30	2.1	42.1					
Borrmann II	145	15.6	40.7					
Borrmann III	614	65.9	34.2					
Borrmann IV	52	5.6	13.7					
Lauren grade				0.003				0.218
Intestinal	455	48.8	50.4		38	42.2	86.5	
Diffuse	477	51.2	37.1		52	57.8	73.1	
Histologic type				0.334				0.263
Mixed-type	272	29.2	40.3		37	41.1	61.3	
Well-differentiated	61	6.5	57.9		7	7.8	83.7	
Medium-differentiated	112	12.0	43.7		8	8.9	53.9	
Poorly-differentiated	365	39.2	37.5		34	37.8	40.2	
Mucinous	58	6.2	38.6		1	1.1	100.0	
Signet-ring cell	29	3.1	47.8		3	3.3	33.3	
Undifferentiated	30	3.2	21.4		0	0.0	0.0	
Squamous cell	5	0.5	25.0		0	0.0	0.0	
Histologic type 2				0.504				0.958
Differentiated	380	40.8	37.6		59	65.6	83.1	
Undifferentiated	552	59.2	40.5		31	34.4	80.6	
Histologic type 3				0.032				0.016
Singal-type	684	73.3	39.3		56	62.2	89.1	
Mixed-type	246	26.4	39.3		34	37.8	72.4	
Histologic Grading				<0.001				<0.001
1	63	6.8	82.5		30	30.0	93.3	
2	352	37.7	62.0		45	50.0	88.6	
3	399	42.8	26.2		17	18.9	78.6	
4	118	12.6	6.8		1	1.1	0.0	
T Stage				<0.001				
T1	90	9.7	82.2					
T2	185	19.8	50.8					
T3	418	44.8	36.5					
T4	239	25.6	19.0					
N Stage				<0.001				0.06
N0	288	30.9	63.8		65	72.2	86.2	
N1	168	18.0	43.7		13	14.4	76.9	
N2	189	20.3	34.0		10	11.1	70.0	
N3	287	22.5	12.2		2	2.2	50.0	
Lymphovascular invasion				0.032				
Negative	208	22.3	40.7					
Positive	724	77.7	22.6					
Peritoneal metastasis				0.022				
Absent	889	88.9	39.8					
Present	43	11.1	19.5					
Hepatic metastasis				0.014				
Absent	878	90.3	38.7					
Present	54	9.6	16.8					

**Figure 2:**
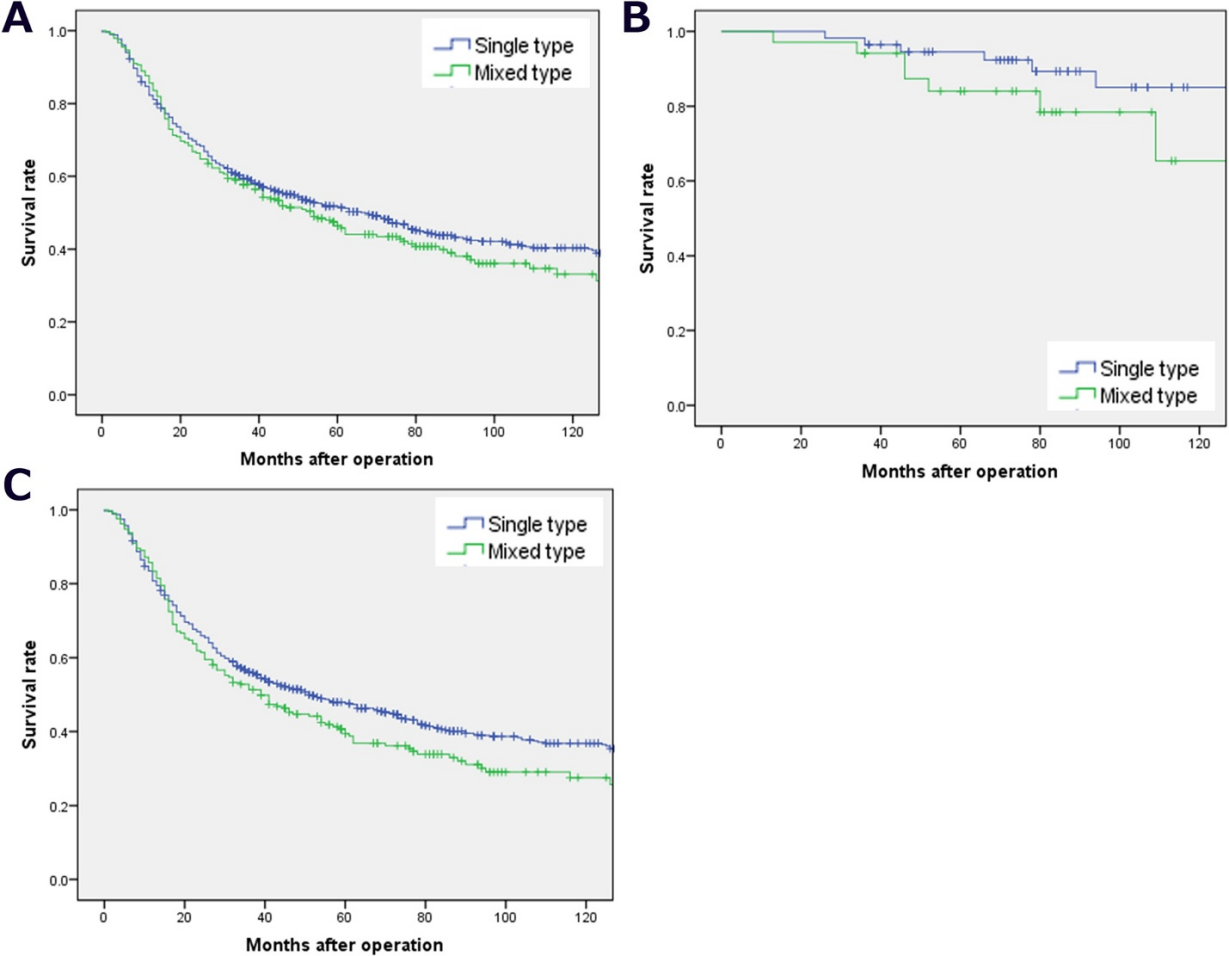
**The 5-year survival rates of gastric cancer patients with mixed form were lower than those with pure form, especially for early stage. (A)** Survival rates of all gastric cancer patients with mixed form were 48.2% and single form were 53.3%, P = 0.305. **(B)** Survival rates of early gastric cancer patients with mixed form were 83.5% and single form were 95.8%, P < 0.01. **(C)** Survival rates of early gastric cancer patients with mixed form were 40.8% and single form were 50.0%, P = 0.037.

Difference of survival reflected by tumor-related factors such as tumor size, tumor depth, lymph node metastasis was shown in Table  3. Compared to the pure histological type, mixed histological type gastric cancer was significant associated with tumor size, lymph node metastasis and depth of invasion (all P < 0.05). All the gastric cancer patients were stratified by advanced and early stage. The 5-year survival rates of advanced and early gastric cancer patients with mixed form were 40.8% and 83.5% respectively, which were lower than those with pure form (50.0% and 95.8%, P < 0.01) ( Figure  2).

**Table 3:** **Relationship between the prognostic factors and histological types for patients with gastric cancer**

Variables	Cases 932	Pure type 684 (73.61)	Mixed type 246 (26.39)	χ2	P value
Gender				0.532	0.466
Male	669	488	181		
Female	263	198	65		
Age(years)				0.039	0.843
≤65	533	391	142		
>65	399	295	104		
Size				<0.001	0.983
≤4 cm	224	165	59		
>4 cm	708	521	187		
Location				0.081	0.96
Lower	672	495	178		
Middle	99	103	38		
Upper	115	88	30		
Entire	46				
Macroscopic Type				11.173	0.018
Early stage	90	66	34		
Borrmann I	30	13	7		
Borrmann II	145	119	26		
Borrmann III	614	453	161		
Borrmann IV	52	35	17		
Lauren grade				0.189	0.003
Intestinal	455	368	128		
Diffuse	477	318	118		
T Stage				9.629	<0.001
T1	90	56	34		
T2	185	140	45		
T3	418	321	97		
T4	239	169	70		
N Stage				3.043	0.551
N0	288	213	75		
N1	168	131	37		
N2	189	135	54		
N3	287	207	80		
Lymphovascular invasion				8.794	0.038
Negative	208	378	121		
Positive	724	300	124		
Peritoneal metastasis				10.322	<0.001
Absent	889	672	215		
Present	43	12	31		
Hepatic metastasis				11.523	<0.001
Absent	878	667	209		
Present	54	17	37

**Figure 3:**
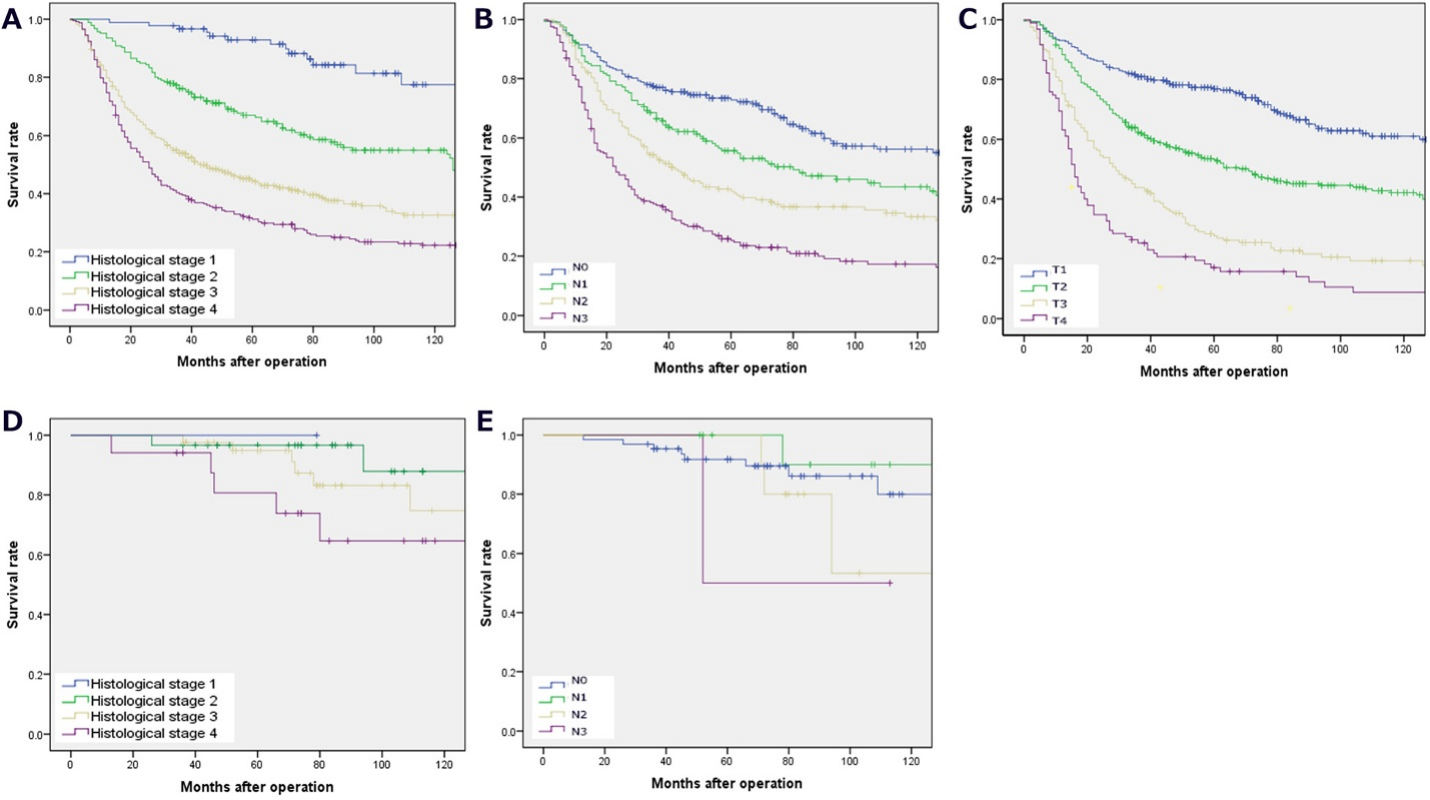
**The 5-year survival rates of gastric cancer patients stratified by histological grading. (A)** showed there is no superiority compared to N stage **(B)** and T stage **(C)**, but the 5-year survival rates of patients with early stage stratified by histological grading **(D)** was much better than N stage **(E)** in predicting the prognosis of the early stages, P < 0.01. Significant difference could be observed in survival curves between the early and advanced stage for the same patients, respectively.

Patients stratified by Histological grading of 214 (55.9%), 182 (28.4%), 492 (15.7%) and 44 (28.4%) defined Histological stage I, II, III and IV, respectively. Statistically significant difference with stratification of early and advanced stage could be observed between patients with the histological grading score, respectively. Mixed histological type gastric cancer had worse prognosis in early and advanced stage (Table  2). No significant difference was observed regarding gender and age of the patients. Significant correlation was observed between H grading scores and all of the tumor-related factors (Table  4).The T, N, and TNM stage are the best classification system to classify overall survival of patients with gastric carcinoma, however, there is no effective methodology to evaluate the prognosis of early stage. The 5-year survival rates of gastric cancer patients stratified by histological grading showed there is no superiority compared to T stage and N stage, but the 5-year survival rates of patients with early stage stratified by histological grading was much better than N stage in predicting the prognosis of the early stages, P < 0.01. Significant difference could be observed in survival curves between the early and advanced stage for the same patients, respectively (Figure  3).

**Table 4:** **Comparison of relationship between Histological grading and prognositic factors**

	Histological grading				P
Variables	H1 (≤2)	H2 (2–3)	H3 (3–4)	H4 (4–5)	
Size					<0.001
≤4 cm	61	39	117	7	
>4 cm	153	143	375	37	
Location					
Lower	150	130	358	35	
Middle	34	25	76	6	
Upper	30	27	58	3	
Entire					
Macroscopic Type					<0.001
Early stage	31	18	49	2	
Borrmann I	10	2	7	1	
Borrmann II	43	33	63	6	
Borrmann III	127	123	334	30	
Borrmann IV	3	6	38	5	
Lauren grade					<0.001
Intestinal	169	127	194	6	
Diffuse	45	55	298	38	
Histologic type					<0.001
Differentiated	164	141	72	3	
Un-differentiated	50	41	420	41	
Depth of tumor					<0.001
T1	30	17	42	1	
T2	46	36	95	8	
T3	92	89	223	14	
T4	46	40	132	21	
Node metastasis					<0.001
N0	87	55	137	9	
N1	42	37	83	6	
N2	44	40	96	9	
N3	41	50	176	20	
Lymphovascular invasion					<0.001
Negative	145	104	240	10	
Positive	77	76	249	35	
Peritoneal metastasis					
Absent	889				
Present	43				
Hepatic metastasis					
Absent	878				
Present	54

The multivariate Cox stepwise proportional hazard model identified Macroscopic type (HR, 1.226, P <0.001), Histological grading (HR, 1.316, P <0.001), T stage (HR, 1.340, P <0.001), N stage (HR, 1.425, P <0.001) as independent predictors of prognosis. In the early gastric cancer, Histological grading (HR, 1.533, P <0.001), Tumor size (HR, 1.412, P <0.001) and N stage (HR, 1.213, P <0.001) as independent predictors of prognosis, histological grading were proved to be the highest HR (Table  5).

**Table 5:** **Mutivariate analysis of the prognostic factors for patients with gastric cancer in overall and early stage**

Variables	Overall			Early stage		
	HR	95% CI	P value	HR	95% CI	P value
Size	1.220	0.958-1.554	0.107	1.412	1.026-1.545	<0.001
Macroscopic type	1.226	1.086-1.385	<0.001	1.365	0.922-1.463	0.103
Lauren grade	1.181	0.894-1.076	0.684	1.133	0.754-1.042	0.725
Histologic type	1.136	0.764-1.352	0.722	1.136	0.764-1.352	0.722
Histologic grading	1.316	1.182-1.465	<0.001	1.533	0.838-1.734	<0.001
N Stage	1.425	1.328-1.529	<0.001	1.213	1.096-1.416	0.036
T Stage	1.340	1.181-1.522	<0.001			
Distant metastasis	1.215	0.935-1.650	0.116			

### Discussion

Generally, tumor stage composed of depth of tumor, lymph node metastasis, and distant metastasis, might account for the most powerful indicator for the prognosis in the majority of malignant tumors [12–15]. Conventional histopathological variables that have been correlated with prognosis of many malignant neoplasms plays an important role in prognostic score, such as Gleason score of prostate cancer, Child-pugh classification of hepatocellular carcinoma, SBR, WHO score of breast cancer [8, 16, 17].

Nevertheless, for gastric cancer, separated from other cancers, the prognostic value of these factors has not been consistently recognized. It has been reported that there is no relationship between gastric histological type and prognosis, there is no comprehensive pathological conditions or anything about gastric histopathological differentiation in the reflection of gastric cancer staging and prognosis [18].

However, the practical value of some of histopathological variables is limited due to complexity of gastric histological composition, coexistence of different malignancy subtypes and ambiguity of tumor biological behaviors [19]. Despite their histological variability, usually one of four patterns predominates. The diagnosis is based on the predominant histological pattern [20]. Histologically, most subtypes of carcinoma occur in early gastric cancer in either pure or mixed forms. We performed this study to compare pure or mixed gastric cancer forms of prognosis, The 5-year survival rates of advanced and early gastric cancer patients with mixed form were 40.8% and 83.5% respectively, which were lower than those with pure form (50.0% and 95.8%, P < 0.01).

Proportion of mixed forms gastric cancer patients with N0 stage were significantly lower than with a pure type, patients of mixed type are more likely to lymph node metastasis. Mixed forms were significantly correlated with T stage, so patients were stratified into early and advanced stage, univariate analysis and survival curve showed 5 -year survival of mixed type of early gastric cancer was significantly lower than that that of a pure type, and there was little difference in the advanced stage.

With the histological grading score, there is significantly different between the 5-year survival curves of H Stage, prognosis of early gastric cancer could be identified obviously, which is not reflected in any other current classification. Indeed, statistically significant difference with so strict stratification was observed between patients with H Stage 1–4.

The T, N, or TNM classification system could exert an excellent ability to classify overall survival of patients with gastric carcinoma also in the current study. However, in early gastric cancers, only small mucosal (<4 cm), superficial (>4 cm) and PenA, PenB may have a low incidence of lymph node metastasis with good prognosis after surgery [21]. There is no effective methodology to evaluate the prognosis of early gastric cancer. Our new histological grading provides a new tragedy to identify the prognosis of early gastric cancer.

Of course, TNM classification system could exert an excellent ability to classify survival of patients with gastric carcinoma also in the current study. Therefore, H grading system newly devised as well as TNM classification system could classify the prognosis of patients with gastric carcinoma with a strict stratification. This score system would provide objective information regarding the outcome of patients treated with curative resection for gastric carcinoma.

In conclusion, H Stage that can be utilized in the majority of the institutes would be quite simple criteria to predict prognosis of gastric carcinoma with a strict stratification.

## Conclusions

The histological score that we developed in the research exert an excellent ability to classify survival of patients with gastric carcinoma, it could classify the prognosis of patients with gastric carcinoma with a strict stratification. The histological score is an independent factor of gastric cancer. It also provides a new strategy and parameter for evaluating the biological behavior and prognosis of gastric cancer.
